# Mechanical Properties of Cu+CuO Coatings Determined by Nanoindentation and Laugier Model

**DOI:** 10.3390/ma18040885

**Published:** 2025-02-18

**Authors:** Sylwia Sowa, Joanna Kacprzyńska-Gołacka, Jerzy Smolik, Piotr Wieciński

**Affiliations:** 1 Łukasiewicz Research Network—Institute for Sustainable Technologies, 6/10 Pułaskiego St., 26-600 Radom, Poland; joanna.kacprzynska-golacka@itee.lukasiewicz.gov.pl (J.K.-G.); jerzy.smolik@itee.lukasiewicz.gov.pl (J.S.); 2 Faculty of Chemistry, Warsaw University of Technology, 3 Noakowskiego St., 00-664 Warsaw, Poland; piotr.wiecinski@pw.edu.pl

**Keywords:** Cu+CuO coatings, fracture toughness, nanoindentation method, Laugier model

## Abstract

Nanoindentation-based fracture toughness measurements of three different materials based on copper oxide with a Berkovich indenter are fascinating topics in material science. The main purpose of this study was to calculate the fracture toughness in mode I (*K_Ic_*) for three copper oxide coatings (Cu+CuO) deposited on a steel substrate by the DC magnetron sputtering method. The parameter *K_Ic_* can be referred to as the critical load (P_critical_), where the cracking process is initiated uncontrollably. The basic mechanical parameters, such as the hardness and Young’s modulus of Cu+CuO coatings, were determined using a Berkovich nanoindenter operated with the continuous contact stiffness measurement (CSM) option. Structural observation was performed by scanning electron microscopy (Helios). Using the nanohardness tester from Anton Paar with a Berkovich diamond indenter with experimentally selected load allowed generation of visible and measurable cracks, which were necessary for *K_Ic_* calculation. Crack lengths were measured by scanning electron microscopy (SEM Hitachi TM3000). The obtained results indicated that the values of hardness and Young’s modulus of Cu+CuO coatings decreased as the power of the magnetron source and the fracture toughness coefficient increased. In the case of the presented study, the Laugier model was chosen for *K_Ic_* determination.

## 1. Introduction

At this time, metal oxide (*MeO*) has attracted much attention because of its unique properties such as being photovoltaic and bactericidal, which have the potential for diverse applications. Based on their bactericidal properties, *MeO* such as AgO, ZnO, and CuO are popular coatings [1,2,3,4,5,6,7,8,9]. From the literature, we know that ZnO has osteogenic potential and stimulates the formation, mineralization, and also the preservation of bone tissue; thus, it is a proper candidate for improving the biological response of the implant surface [10]. Copper oxide can form two stable oxides in two different oxidation states I (Cu_2_O) and II (CuO). Both types of copper oxides can be obtained by several techniques such as reactive sputtering, molecular beam epitaxy, chemical and thermal oxidation, and electroplating. Deposition of selective Cu_2_O thin film is difficult because the formation of CuO is thermodynamically favorable in a reactive environment such as the magnetron sputtering method or molecular beam epitaxy. In this research, CuO attracts special attention because it is the simplest compound from the copper family and indicates several potentially wide-ranging applications in energy harvesting and storage, such as solar cells, photo-electro-chemical cells (PECs), photo-catalysis and lithium-ion batteries [11,12].

All *MeO* can be classified as brittle materials. Most of the studies reported to date have been focused on the physical and photocatalytic properties of CuO thin films [13] and there has been no information about fracture toughness measurement for copper oxide coatings.

For investigation of the mechanical characteristics of coatings and various materials in the submicron and nano-scales, the best technique is nanoindentation. Nanoindentation is a useful tool for evaluating fracture toughness in mode I (*K_Ic_*) of materials based on the formation of cracks at the corners during indentation. The crack lengths generated by pressing the indenter into the tested material can be connected to the fracture toughness coefficient of materials. Generally, nanoindentation is used for measuring mechanical properties such as hardness, modulus, and stiffness [14,15,16]. The literature presents a few interesting methods for determining the fracture toughness of different materials. In 1970, Evans and Charles proposed the nanoindentation method with a Vickers indenter as a technique for identifying the *K_Ic_* coefficient value [17]. For example, the Niihara and Anstis model also concentrated on determining fracture toughness for various types of materials in scope *K_Ic_* = 0.74–12 MPa/m^1/2^ with a Vickers indenter [18,19,20,21]. The investigations by Laugier [22], Dukino [23], and Ouchterlony [24] reformed the Niihara model by presenting a coefficient which concentrates on the geometry of the Berkovich indenter. Our previous investigation confirmed that it may be possible to choose the Laugier model to evaluate the fracture toughness of different coatings [25,26].

This research aimed at presenting the chance of fracture toughness measurement of coatings based on copper oxide by the nanoindentation method with a Berkovich indenter. This article focuses on the characterization of nanomechanical Cu+CuO coatings deposited on steel S600 by means of the direct current magnetron sputtering method (DC-MS) in an inert gas atmosphere at low temperatures without the use of substrate polarization. All deposited Cu+CuO coating structures were specified by scanning electron microscopy (SEM). Hardness and Young’s modulus and fracture toughness of Cu+CuO coatings were measured using Berkovich nanoindentation in continuous stiffness measurement (CSM) mode. Changes in the mechanical properties of Cu+CuO coatings can be discussed in connection with the increase in the power of the magnetron source during the deposition coating process. The authors also analyzed how the magnetron sputtering power influences the mechanical properties of Cu+CuO coatings, which was evaluated using the Laugier model.

## 2. Materials and Methods

### 2.1. Preparation of Cu+CuO Coatings

Cu+CuO coatings with different powers of magnetron source (P_MCu_ = 200 W, 350 W, and 500 W) were prepared on S600 steel substrates. The samples were characterized by the following parameters: diameter of 25 mm, thickness of 6 mm, and surface roughness R*a* ≤ 0.05. The deposition process used the DC-MS (direct current magnetron sputtering) method with the original magnetron system constructed and fabricated by Łukasiewicz Research Network—Institute for Sustainable Technologies in Radom (Standard 3, Łukasiewicz—ITeE Radom). The technological device Standard 3 was equipped with targets of Cu (purity of target was 99.99%) located in the wall chamber. The parameters of the target were as follows: diameter of target d = 100 mm, thickness of target g = 7 mm. The distance between the sample and the plasma source was 200 mm. The processing time was 2 h. First, a thin Cu coating was deposited as an adhesion coating with steel (hence the notation Cu+CuO). This coating was deposited at a reactive gas atmosphere of 100% Ar. The technological processes were made without substrate polarization at low temperature. The power of the magnetron source for the deposition of Cu+CuO was selected experimentally (Table 1).

### 2.2. Mechanical Properties of Tested Coatings

The hardness (H) and Young’s modulus (E) of the analyzed Cu+CuO coatings on steel S600 substrate material were examined with a CSM-TTX/NH2 nanohardness tester (NHT, CSM Instruments, Needham Heights, MA, USA) by Anton Paar equipped with a Berkovich diamond intender, pyramid-shaped with an angle of 65°. The device allows selection of loads within the range 0.05–500 mN and a precise selection of the penetration depth of the indenter in the range of 1000 µm. The maximum penetration depth of the intender was h_max_ < 0.1 (10%) of the total thickness of the coatings. For each of the deposited coatings, the right number of measurements (15 repetitions) of hardness and Young’s modulus were examined. Based on measurements, the plasticity index *H*/*E* and resistance to plastic deformation *H*^3^/*E*^2^ were calculated.

### 2.3. Surface Characterization

The morphology of the surface of all Cu+CuO coatings was verified via images recorded with scanning electron microscopy using a Helios G5 PFIB CX (Thermo Fisher Scientific, Waltham, MA, USA) equipped with a Schotty field emission gun with a UC+monochromator (Thermo Fisher Scientific, Waltham, MA, USA), in-lens and in-column SE/BSE detectors, and an Octane Elite Super (70 mm^2^) EDS detector (Edax, Pleasanton, CA, USA).

The roughness in the micro-scale was investigated by a Form Talysurf PGI 830 profilograph by Taylor Hobson (Radom, Poland), which was equipped with a measuring head with a resolution of 8.0 nm. The measurement was made using a laser sensor which moves along the tested sample. The maximum number of collected points was 1,600,000. The measuring speed range was from 0.1 to 2 mm/s. The profilograph has software that enables both 2D (profiles) and 3D (topography) analysis, and also allows the determination of roughness and waviness parameters. During the measurements, three important parameters were measured: R*a*, R*z*, and R*t*. R*a* is the arithmetic mean from all values of the roughness profile R within the measuring distance l_m_. It describes the average deviation of this surface profile from the mean line. R*t* is the vertical distance between the highest peak and lowest peak of the roughness profile R within the overall measuring distance l_m_. R*z* is the average value from the individual roughness depths of five individual measuring distances in sequence [27]. For better statistics, three measurements were made, and the average values of these measurements were determined.

### 2.4. The Test Method for Fracture Toughness Calculation

In the case of all three tested Cu+CuO coatings, the CSM-TTX/NH2 nanohardness tester (NHT) by Anton Paar, with a diamond Berkovich indenter, was used. The received samples were also analyzed for assessment of fracture toughness *K_Ic_*. The nanohardness tester by Anton Paar equipped with a Berkovich diamond indenter allows generation of visible and measurable cracks. Based on the Laugier formula, the value of the fracture toughness coefficient *K_Ic_* was calculated:

(1) $$K_{Ic}=X_{v}\cdot {\left(\frac{a}{l}\right)}^{\frac{1}{2}}\cdot {\left(\frac{E}{H}\right)}^{\frac{2}{3}}\cdot \frac{P}{c^{\frac{3}{2}}}$$

where *K_Ic_*—the fracture toughness coefficient; *X_v_*—indenter geometry factor (for the Laugier equation, *X_v_* = 0.016); *E*—Young’s modulus (GPa); *P*—the applied load (mN); *a*—the distance between the corner and the center of indentation (µm); *l*1, *l*2, *l*3—the average crack length, *l* = (*l*1 + *l*2 + *l*3)/3, *c = l + a*—the sum of *a* and *l* [22,25,26].

**Figure 1:**
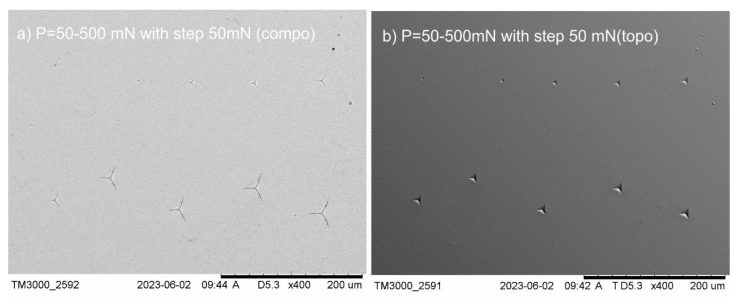
SEM images, for example, Cu+CuO(2) coatings with the selection of the critical indenter load which initiated measurable, detectable cracks in the range 50–500 mN with step 50 mN in (**a**) compo mode and (**b**) topo mode.

The investigation of the brittle cracking for coatings based on copper oxide can be divided into two steps. The first step included the selection of the critical indenter load (*P_critical_*) which led to measurable and visible cracks from the corner of the indentations. To find the appropriate load, indentation experiments were conducted with different applied loads of 50, 100, 150, 200, 250, 300, 350, 400, 450, and 500 mN for each of the examined samples (Figure 1). The critical load (*P_critical_*) which generated visible and measurable cracks was chosen to make 20 indentations, and the values of *a* and *l* (crack lengths) were measured. In accordance with the mathematical principles developed by Laugier, values of fracture toughness coefficient in mode I (*K_Ic_*) were calculated [22,25,26]. The second step was observation and mensuration of crack lengths using scanning electron microscopy SEM with a Hitachi TM3000 (Łukasiewicz-ITeE, Radom, Poland).

## 3. Results

### 3.1. Characterization of Cu+CuO Coatings

This section includes the mechanical results of three Cu+CuO coatings. Before measurement, the surface of all analyzed coatings was distinguished by high smoothness and good coherence, free of cracks and defects (R*a* ≤ 0.05). The thickness of the tested coatings amounted accordingly to 1.75 µm, 2.00 µm, and 3.70 µm. The measurement of hardness was realized according to the basic rule of 10% of the whole thickness of coatings, respectively, 170 nm for Cu+CuO(1), 200 nm for Cu+CuO(2), and 351 nm for Cu+CuO(3). The characteristic mechanical properties of the studied Cu+CuO coatings are listed in Table 2.

### 3.2. Structure Characterization

**Figure 2:**
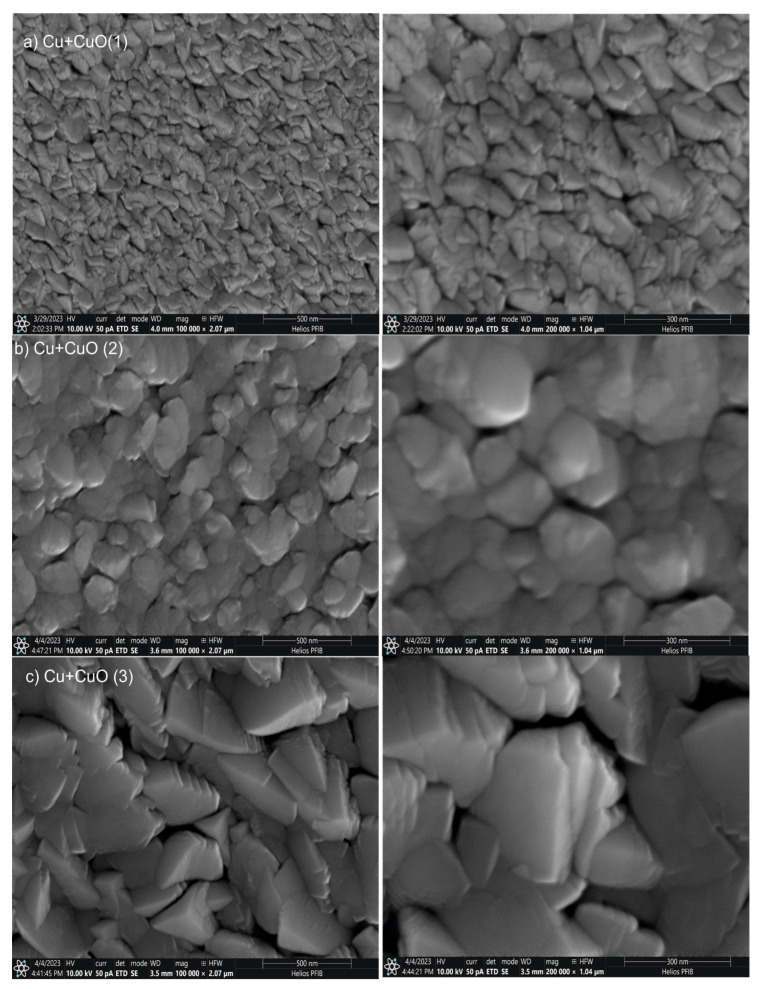
SEM images from the surface for a group of Cu+CuO coatings deposited with different powers of magnetron source: (**a**) Cu+CuO (200 W), (**b**) Cu+CuO (350 W), and (**c**) Cu+CuO (500 W).

The SEM observation of the steel S600 surface with three types of Cu+CuO coatings is shown in Figure 2. All tested coatings are characterized by visible grains. The size of the individual grain changes (increases) as the magnetron power increases. For Cu+CuO(1) coating deposited with a magnetron power of 200 W, a fine structure with grains with sharp edges was observed. Cu+CuO(2) coating deposited with 350 W showed larger, non-equiaxed, non-homogenous grains (large grain size distribution). For Cu+CuO(3) coating deposited with a power of magnetron source 500 W, grain disturbances (sharp edges)related to the direction of their growth and irregular surface morphology could be observed. The fracture toughness coefficient and roughness parameters of these coatings can be related. It can be also observed that an increase in magnetron power leads to an increase in grain size (Figure 2) and increase in coating thickness (Table 2).

A summary of the roughness parameters R*a*, R*z*, and R*t* for tested Cu+CuO coatings is presented in Table 3. The highest value of R*a* parameters was observed for Cu+CuO(3). For Cu+CuO(1) coatings, the value of the R*a* parameter is four times lower than for Cu+CuO(1). In the case of R*z* parameters, the lowest value is for Cu+CuO(1) but the highest is for Cu+CuO(2). Cu+CuO(1) coating showed the R_t_ parameter with a lower value, 0.038 um, but the highest similar to R*_z_* for Cu+CuO(2). The roughness R*a* increases with grain size from Cu+CuO(1) to Cu+CuO(3).

### 3.3. Calculation Method of K_Ic_ for Cu+CuO Coatings

**Figure 3:**
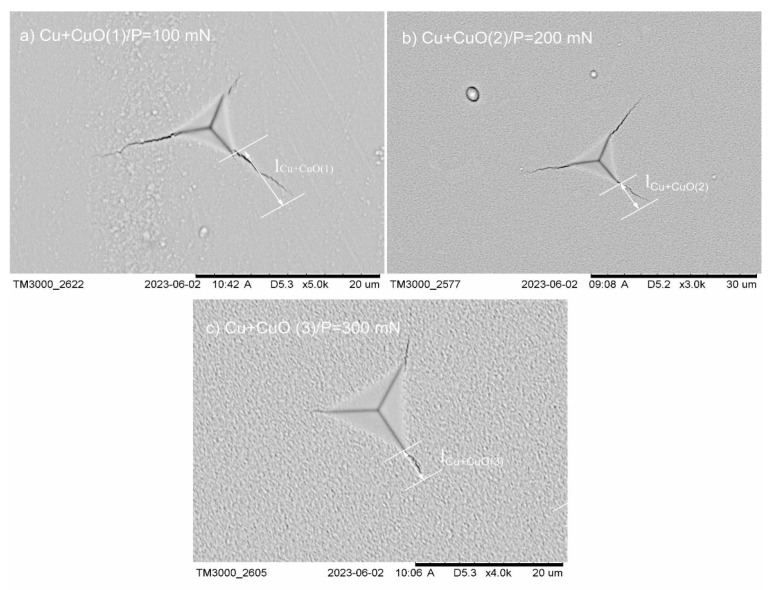
Representative SEM images with detectable indentations as a result of using different values of applied load for coatings: (**a**) Cu+CuO(1), *P_critical_* = 100 mN; (**b**) Cu+CuO(2); *P*_critical_ = 200 mN; (**c**) Cu+CuO(3); *P_critical_* = 300 mN.

The choosing of the critical load (*P_critical_*) which generated measurable cracks during the section of the five values of applied loads 100, 200, 300, 400, and 500 mN for Cu+CuO(2) and Cu+CuO(3) analyzed coatings was the main step of the measurement procedure. For Cu+CuO(1) coating, finding the critical load (*P_critical_*) was much trickier, because the applied load, ranging from 100 to 500 mN, was too large and destroyed the coating. The range of applied load for Cu+CuO(1) was from 50 mN to 125 mN with every step being 25 mN. After selection by the nanohardness tester (NHT), imaging of all generated indentations with cracks was performed using scanning electron microscopy (SEM-Hitachi TM3000). The SEM images of indentations for different loads showed that the critical indenter load *P_critical_* = 100 mN led to generating of detectable and well-measurable cracks from the corner of the indentations for Cu+CuO(1) coating. In the case of Cu+CuO(2) coating, the critical load was 200 mN. An interesting situation was observed for Cu+CuO(3), where generation of visible cracks needed an applied load of 300 mN (Figure 3a–c).

The accepted methodology requires the performance of 20 indentations for all selected Cu+CuO coatings. The penetration depth of Cu+CuO(1) was h_m100mN_ = 1000 nm. For Cu+CuO(2) coating, the penetration depth was h_m200mN_ = 1400 nm, and for Cu+CuO(3) coating the penetration depth was, respectively, h_m300mN_ = 1800 nm. The next step was to measure the average values of *l* and *a*, which can be presented according to the following Equations:

(2) $$a=\left(a_{n=1}+a_{n=2}+\cdots +a_{n=20}\right)/20$$

(3) $$l=(l_{n=1}+l_{n=2}+\cdots +l_{n=20})/20$$

**Figure 4:**
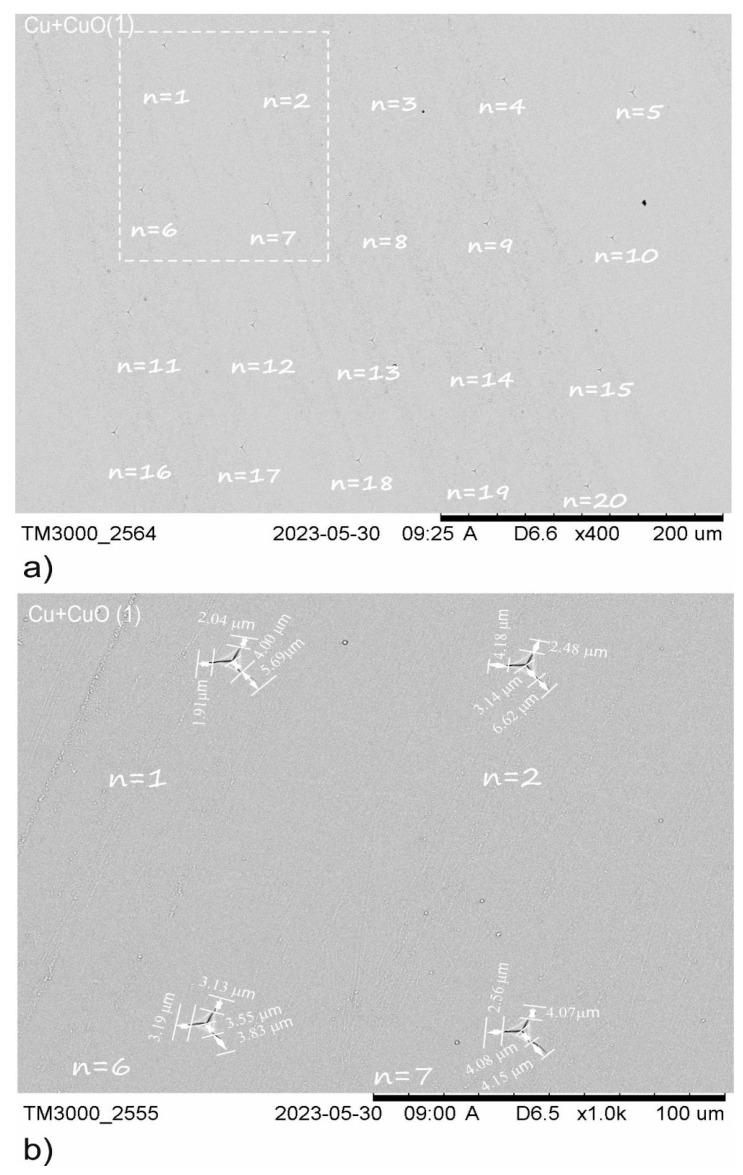
Illustration of SEM images for Cu+CuO(1) coating used for K_Ic_ coefficient calculation. (**a**) Group of 20 indentations at a critical load *P_critical_* = 100 mN; (**b**) example of crack lengths for selected indentations, where *n* = 1, 2, 6, 7.

**Figure 5:**
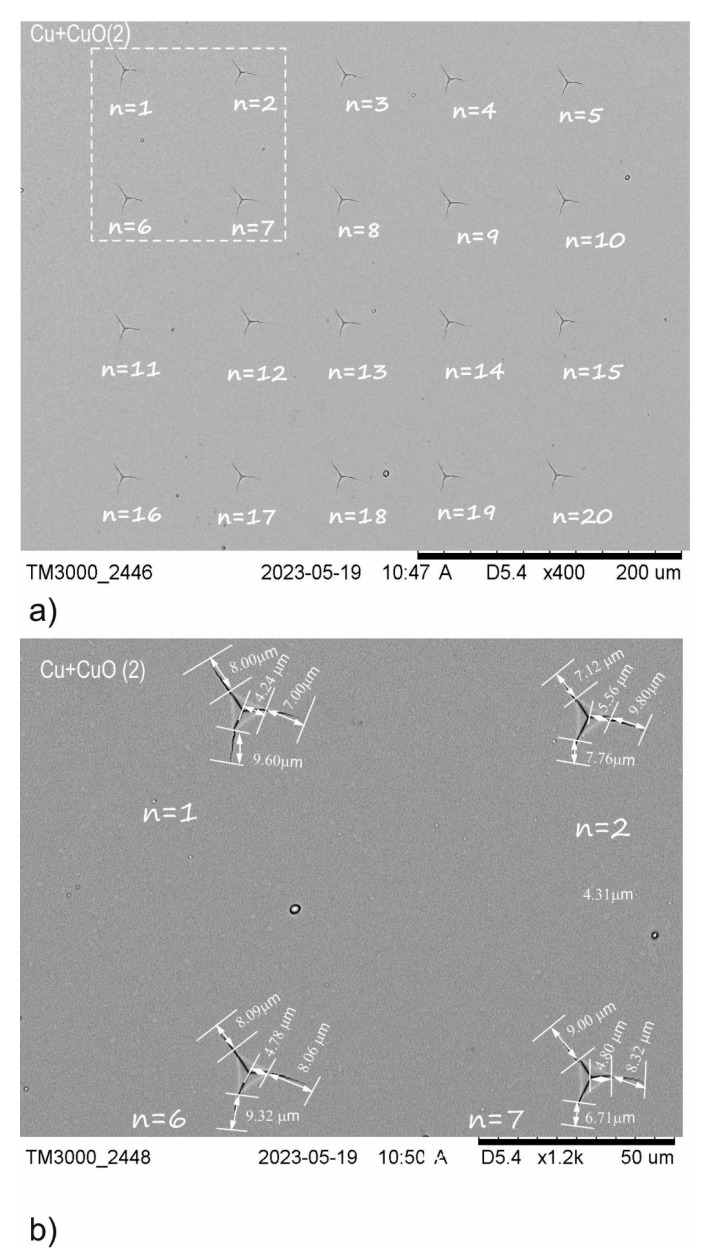
Illustration of SEM images for Cu+CuO(2) coating used for K_Ic_ coefficient calculation. (**a**) Group of 20 indentations at a critical load *P_critical_* = 200 mN; (**b**) example of crack lengths for selected indentations, where *n* = 1, 2, 6, 7.

**Figure 6:**
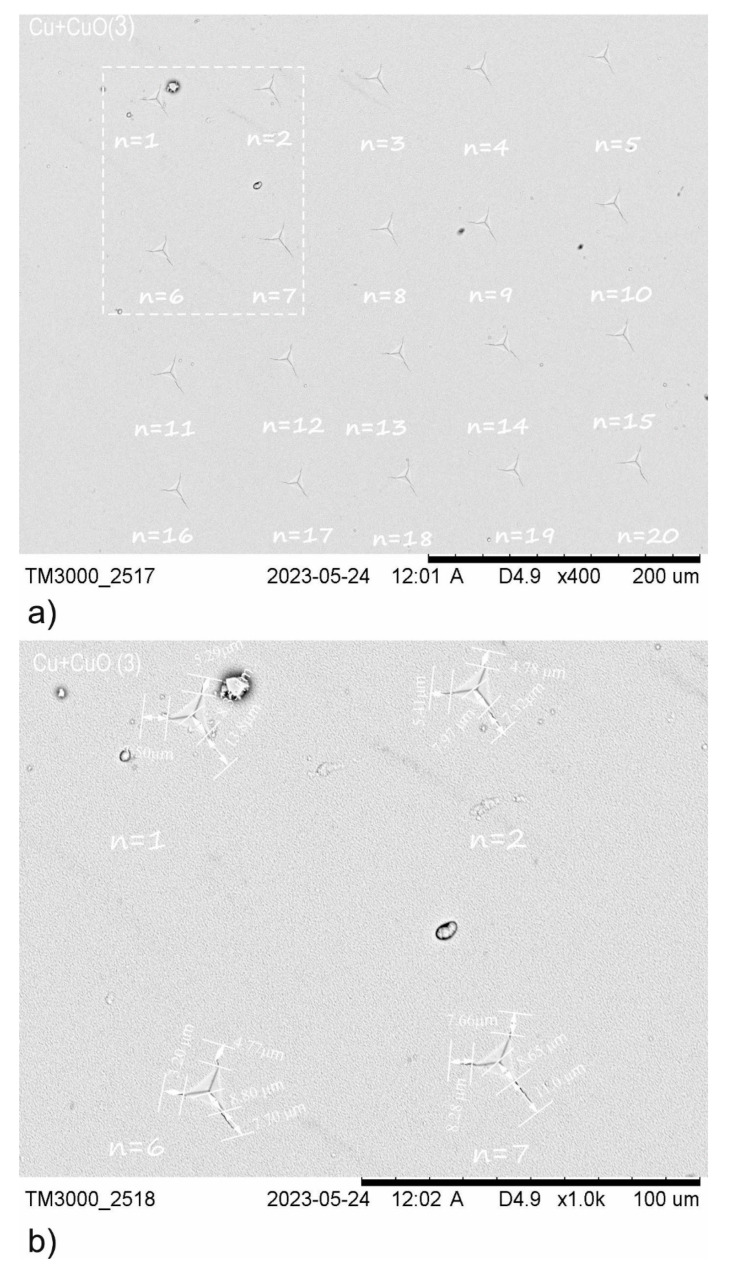
Illustration of Cu+CuO(3) coating used for K_Ic_ coefficient calculation. (**a**) Group of 20 indentations at a critical load P_critical_ = 300 mN; (**b**) example of crack lengths for selected indentations, where *n* = 1, 2, 6, 7.

The average values of *l* and *a* were calculated following Equations (2) and (3), for the complete series of 20 indentations. Figure 4, Figure 5 and Figure 6 show that increasing the magnetron power source and hence the thickness of the Cu+CuO series effects a change in the average crack lengths (*l*) and distance between the corner and middle of indentation (*a*). For Cu+CuO(1), the *l* value is 3.42 µm, for Cu+CuO(2) the *l* value is 5.13 µm, and for Cu+CuO(3), the *l* value is 7.11 µm. The crack lengths for Cu+CuO(3) were approximately two times higher than for Cu+CuO(1). The second observation is about the length of the *a* value. The highest average value of *a* = 8.75 ± 1 µm for Cu+CuO(2) but the lowest average value of *a* = 4.69 ± 2 µm, which was presented for Cu+CuO(1), and it is around two times lower.

## 4. Discussion

The completed research using the nanoindentation method, especially the Laugier model, showed that a change in power of the magnetron sputtering source affects the thickness and fracture toughness values of all investigated coatings. This phenomenon can explain why modifying the power magnetron during the process can influence the plasma density and chamber pressure. It can affect the structure of deposited coatings. It was also observed that crack lengths were changed.

**Figure 7:**
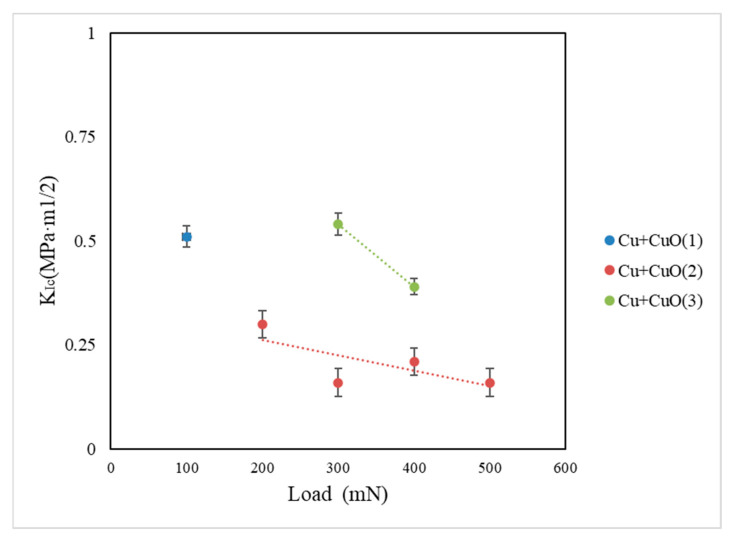
Fracture toughness *K_Ic_* analysis for three Cu+CuO coatings depending on the applied load (P = 100, 200, 300, 400 and 500 mN).

In this study, the authors showed how the mechanical properties of three Cu+CuO coatings change with magnetron power. Cu+CuO(1) coating deposited with 200 W of magnetron power source presents the highest hardness H = 3.40 ± 0.2 GPa and Young’s modulus E= 88.2 ± 4 GPa values. The plasticity index of this coating H/E is 0.038, and resistance to plastic deformation of coatings H^3^/E^2^ shows the lowest value, 0.005. The fracture toughness coefficient gives a value of *K_Ic_* = 0.51 MPa ·m^1/2^ at the critical load *P_critical_* = 100 mN. For comparison, Cu+CuO(3) coatings prepared with 500 W of magnetron power show the lowest value of hardness H = 2.20 ± 0.2 GPa and Young’s modulus E = 36 ± 2 GPa. The plasticity index of these coatings H/E is 0.061, and resistance to plastic deformation of coatings H^3^/E^2^ exhibits the lowest value, 0.008. The fracture toughness coefficient achieves the value of *K_Ic_* = 0.54 MPa·m^1/2^ at the highest *P_critical_* 300 mN compared to other coatings. The very interesting observations are for Cu+CuO(2) coatings created with 350 W of magnetron power, because the hardness H = 3.14 ± 0.2 GPa and Young’s modulus E = 51 ± 4 GPa values are in the middle of all values but the resistance to plastic deformation of coatings H^3^/E^2^ provides the highest values 0.012 from all coatings. For Cu+CuO(2) coating, the fracture toughness coefficient is *K_Ic_* = 0.30 MPa·m^1/2^ in the critical load *P_critical_* = 200 mN is the lowest value from all coatings (Figure 7). It can be observed that the value of *K_Ic_* = 0.16 MPa·m^1/2^ for Cu+CuO(2) is 3.5 times lower at *P_critical_* = 300 mN compared to *K_Ic_* = 0.54 MPa·m^1/2^ for Cu+CuO(3). In the Cu+CuO(2) and Cu+CuO(3) coatings, values of fracture toughness coefficient decrease when increasing the applied load. In Cu+CuO(2) coating, it is possible to generate visible and measurable cracks at an applied load of 500 mN. In the case of Cu+CuO(3), the applied load of 500 mN destroyed the coating. These phenomena can be explained by differences in morphology and sizes of grain, which were observed for all deposited coatings [28]. In recent years, the effect of grain size on fracture toughness has been analyzed [29,30,31]. A smaller average grain size results in greater fracture toughness values [30,32]. Chaudhuri [33] concluded that the effect of grain size on fracture toughness mainly depends on the fracture mode. When the fracture mode was inter-granular, fracture toughness decreased with increasing grain size, while when the fracture mode was quasi-cleavage, fracture toughness was unaffected by grain size. Therefore, we can consider that grain size may have a rather complex effect on fracture toughness.

**Figure 8:**
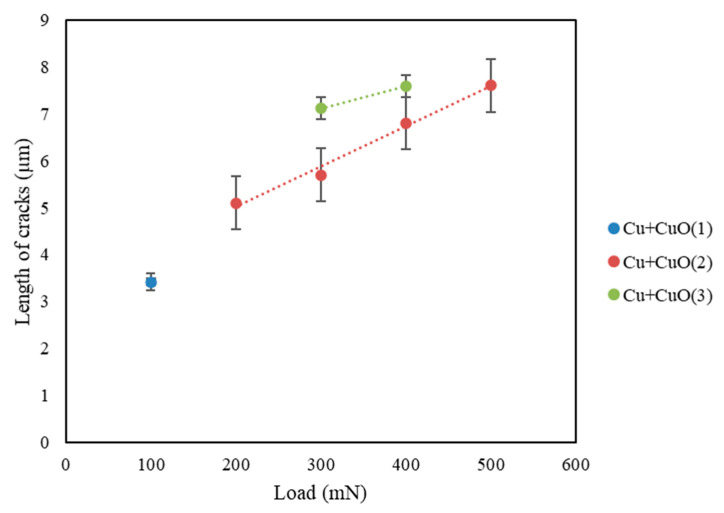
The applied load (*P*) vs. crack length (*l*) ratio for selected indentations of our Cu+CuO coatings.

Figure 8 shows the variation in the length of cracks (*l*) at various loads (*P*) for selected samples where detectable, measurable cracks were initiated. In the case of Cu+CuO(1), where the generation of visible cracks was not easy, only one value, of 100 mN, was collected and it is *P_critical_*. This coating was the thinnest of all, and applying a higher load than 100 mN destroyed coatings. In the case of Cu+CuO(2) and Cu+CuO(3), there were more load values which could generate visible and measurable cracks. The generated cracks were chosen for 200 mN, 300 mN, 400 mN, and 500 mN in the case of Cu+CuO(2). The results for Cu+CuO(3) coating deposited with the power of magnetron source 500 W confirmed that we have brittle materials. The measurable cracks were observed only for 300 mN and 400 mN. The applied load of 500 mN is too high and destroyed this coating. The obtained results confirmed that the magnetron power affects grain growth. Grain growth reduces the hardness and brittleness of tested coatings. It can be concluded that the phase structure and morphology of a series of Cu+CuO coatings impact the cracking mechanics of the coatings. A similar situation compared to Cu+CuO(2) was reported in the literature [34]. The authors in the article [34] showed that changing deposition parameters leads to changing dominating phase structure. It is possible to create Cu_2_O phase characterized by spherical grains such as in Cu+CuO(2) coating. It can also be shown that crack lengths increase with applied load for Cu+CuO(2) and Cu+CuO(3).

## 5. Conclusions

In summary, a combination of SEM and nanoindentation techniques have been carried out to investigate the morphology and nanomechanical properties of Cu+CuO coatings deposited on S600 steel using direct current magnetron sputtering (DC-MS) with three different powers of magnetron sources. All tested coatings have a hardness ranging from 3.40 ± 0.2 GPa to 2.20 ± 0.2 GPa and Young’s modulus ranging from 88.2 ± 4 GPa to 36 ± 4 GPa. The decrease in hardness can be mainly attributed to the effect of surface morphology and grain size. Experimental results of fracture toughness measurement for the series of Cu+CuO coatings confirmed the possibility of generating visible cracks. The study showed that crack initiation for the Cu+CuO coatings appeared when the critical load (*P_critical_*) was operated on the Berkovich indenter. The range of the applied load on the indenter was selected for all coatings as follows, i.e., *P* = 50–125 mN for Cu+CuO(1) and *P* = 100–500 mN for the other coatings (Cu+CuO(2) and Cu+CuO(3)). Three parameters can influence the fracture toughness coefficient: thickness, phase structure, and morphology. The deposition conditions can significantly change the brittleness of coatings. The research showed that it is very convenient to use the nanoindentation method to assess the fracture toughness coefficient K_Ic_ of coatings based on copper oxide. It can be concluded that the Laugier model gives constant fracture toughness values and can be a sufficient method to assess the brittleness of oxide coatings. In the case of the presented coatings, a more extensive analysis of the scientific problem which is mechanical properties will be required in the future.

## Figures and Tables

**Table 1:** The deposition parameters for the series of Cu+CuO coatings deposited by DC-MS method.

Coatings	Atmosphere	Pressure (Pa)	U_Bias_ (V)	Power of Cu Magnetron Source (W)
Cu+CuO(1)	Ar 90% + 10% O_2_	0.5	0	200
Cu+CuO(2)			0	350
Cu+CuO(3)			0	500

**Table 2:** The thickness, critical load, hardness, Young’s modulus, H/E and H^3^/E^2^, and the characteristic parameters which were used for the calculation of the K_Ic_ coefficient for Cu+CuO coatings.

Coatings	Thickness (µm)	Critical Load *P_critical_* (mN)	Hardness H (GPa)	Young’s Modulus E (GPa)	Plasticity Index H/E	Resistance to Plastic Deformation H^3^/E^2^	*a* (μm)	*l* (μm)	*K_Ic_* (MPa*m^1/2^)
Cu+CuO(1)	1.75	100	3.40 ± 0.2	88.2 ± 4	0.038	0.005	4.69 ± 2	3.42 ± 0.7	0.51 ± 0.2
Cu+CuO(2)	2.00	200	3.14 ± 0.2	51 ± 4	0.061	0.012	8.70 ± 1	5.08 ± 2	0.30 ± 0.05
Cu+CuO(3)	3.70	300	2.20 ± 0.2	36 ± 2	0.061	0.008	7.28 ± 2	7.11 ± 1	0.54 ± 0.1

**Table 3:** The roughness parameters R*a*, R*z*, and R*t* for a series of Cu+CuO coatings deposited by the DC-MS method.

Coatings	R*a* (µm)	R*z* (µm)	R*t* (µm)
Cu+CuO(1)	0.002	0.025	0.038
Cu+CuO(2)	0.003	0.193	0.543
Cu+CuO(3)	0.008	0.127	0.289

## Data Availability

The original contributions presented in the study are included in the article, further inquiries can be directed to the corresponding author.
